# Synergistic Actions of Hematopoietic and Mesenchymal Stem/Progenitor Cells in Vascularizing Bioengineered Tissues

**DOI:** 10.1371/journal.pone.0003922

**Published:** 2008-12-15

**Authors:** Eduardo K. Moioli, Paul A. Clark, Mo Chen, James E. Dennis, Helaman P. Erickson, Stanton L. Gerson, Jeremy J. Mao

**Affiliations:** 1 Columbia University Medical Center, Tissue Engineering and Regenerative Medicine Laboratory (TERML), College of Dental Medicine, New York, New York, United States of America; 2 Department of Neurological Surgery CSC, University of Wisconsin at Madison Hospital, Madison, Wisconsin, United States of America; 3 Department of Orthopaedics, Case Western Reserve University, Cleveland, Ohio, United States of America; 4 Case Comprehensive Cancer Center, Case Western Reserve University, Cleveland, Ohio, United States of America; University of Michigan, United States of America

## Abstract

Poor angiogenesis is a major road block for tissue repair. The regeneration of virtually all tissues is limited by angiogenesis, given the diffusion of nutrients, oxygen, and waste products is limited to a few hundred micrometers. We postulated that co-transplantation of hematopoietic and mesenchymal stem/progenitor cells improves angiogenesis of tissue repair and hence the outcome of regeneration. In this study, we tested this hypothesis by using bone as a model whose regeneration is impaired unless it is vascularized. Hematopoietic stem/progenitor cells (HSCs) and mesenchymal stem/progenitor cells (MSCs) were isolated from each of three healthy human bone marrow samples and reconstituted in a porous scaffold. MSCs were seeded in micropores of 3D calcium phosphate (CP) scaffolds, followed by infusion of gel-suspended CD34^+^ hematopoietic cells. Co-transplantation of CD34^+^ HSCs and CD34^−^ MSCs in microporous CP scaffolds subcutaneously in the dorsum of immunocompromized mice yielded vascularized tissue. The average vascular number of co-transplanted CD34^+^ and MSC scaffolds was substantially greater than MSC transplantation alone. Human osteocalcin was expressed in the micropores of CP scaffolds and was significantly increased upon co-transplantation of MSCs and CD34^+^ cells. Human nuclear staining revealed the engraftment of transplanted human cells in vascular endothelium upon co-transplantation of MSCs and CD34^+^ cells. Based on additional *in vitro* results of endothelial differentiation of CD34^+^ cells by vascular endothelial growth factor (VEGF), we adsorbed VEGF with co-transplanted CD34^+^ and MSCs in the microporous CP scaffolds *in vivo*, and discovered that vascular number and diameter further increased, likely owing to the promotion of endothelial differentiation of CD34^+^ cells by VEGF. Together, co-transplantation of hematopoietic and mesenchymal stem/progenitor cells may improve the regeneration of vascular dependent tissues such as bone, adipose, muscle and dermal grafts, and may have implications in the regeneration of internal organs.

## Introduction

Poor angiogenesis is a common and critical barrier in tissue regeneration. Regenerating tissue over 100–200 µm exceeds the capacity of nutrient supply and waste removal by diffusion, and thus requires an intimate supply of vascular networks [1], [2]. Previous attempts in engineered angiogenesis have relied on the delivery of angiogenic growth factors, transplantation of proangiogenic cells or the fabrication of blood vessel analogs [3]–[6]. In a number of meritorious studies, angiogenesis in scaffolding materials has been induced by a number of angiogenic cytokines such as vascular endothelial growth factor (VEGF), platelet derived growth factors (PDGF), and basic fibroblast growth factor (bFGF) [7]–[14]. Despite promising results, there are continuing concerns over the cost of multiple cytokines and delivery, potential toxicity, and suboptimal endothelial migration in large tissue grafts. The transplantation of proangiogenic cells, such as endothelial cells or endothelial progenitor cells, has led to the formation of blood vessels with suboptimal life span [15]. Short of sustained survival of transplanted endothelial cells, neovasculature fails to recruit the obligatory perivascular cells including mural cells, and does not resemble native, multilayered mature microvessels. Despite tremendous progress, the field of angiogenesis is viewed as top priority in tissue regeneration and tissue engineering, and also the area of least progress in the past decade [16].

Bone marrow is populated by heterogeneous cell types including end-lineage cells, committed tissue progenitors, and multipotent stem/progenitor cells [17], [18]. Two multipotent stem/progenitor cells can be readily isolated from a single bone marrow aspirate: mesenchymal stem/progenitor cells (MSCs) and hematopoietic stem/progenitor cells (HSCs) [17], [18]. Previous work has well explored the regeneration of mesenchymal phenotypes such as bone, adipose and cartilage tissues, by MSCs [19]. During development, mesenchymal progenitor cells co-localize in hematopoietic sites and act as stromal support for tissue homeostasis [20]. During endochondral bone development, invasion of the primary ossification center artery precedes bone formation [21]. Hypertrophic chondrocytes express several critical transcriptional factors and cytokines, including the pivotal vascular endothelial growth factor (VEGF), and elaborate angiogenesis, which in turn promotes bone formation [22]. During bone fracture healing, some of the mobilized repair cells are vascular derived and migrate into the fracture site to participate in the healing process [23]–[25]. The rate of fracture healing is related to angiogenesis [26], [27]. For instance, poor bone healing after irradiation is largely attributed to a compromised vascular bed; conversely, enhancement of vascular supply promotes the regeneration of irradiated bone [28]. Whereas it is logical to exploit the full potential of MSCs on bone regeneration, suboptimal vascularization, a commonly recognized barrier of bone tissue engineering, has not been addressed by taking advantage of the capacity for neovascularization and hematopoiesis by HSCs.

Recently, a great deal of interest has focused on the interactions between HSCs and MSCs [29]. For example, HSCs promote osteogenic differentiation of MSCs via niche-initiated pathways *in vitro* [30]. The mechanism of this interplay between HSCs and MSCs is believed to follow the expression of bone morphogenetic protein 2 (BMP-2) and BMP-6. Conversely, osteoblasts facilitate the mobilization of hematopoietic stem cells [31]. Interestingly, peripheral blood CD34^+^ cells differentiate into cells that express osteogenic markers such as osteocalcin and may participate directly in bone healing [32]. During early development, CD34^+^ haemagioblasts have been manipulated for their potential to differentiate into vascular progenitor cells [30]. However, little is known whether co-transplantation of HSCs and MSCs regenerates vascularized tissues including bone. CD34^+^ hematopoietic and CD34^−^ mesenchymal stem/progenitor cells are co-inhabitants of bone marrow, but have rarely been applied in conjunction to heal tissue defects. In the present study, we co-transplanted bone marrow derived human MSCs and HSCs in the micropores of 3D calcium phosphate (CP) scaffolds. Following the delivery of MSCs to the micropores of CP scaffolds, HSC-seeded Matrigel was infused into MSC-residing micropores. Four weeks after ectopic implantation in immunodeficient mice, human HSC and MSC co-seeded grafts yielded marked vascular number and diameter, and increased human osteocalcin expression, in comparison to MSC transplantation alone. We then observed that VEGF stimulated HSCs to differentiate into endothelial-like cells, which expressed von Willebrand factor and formed intercellular tubular structures *in vitro*. We subsequently delivered VEGF to MSC- and HSC-co-transplanted microporous CP scaffolds *in vivo*. The average vessel number and diameter upon VEGF delivery in MSC- and HSC-seeded microporous CP scaffolds further increased. Due to their co-localization in bone marrow and therefore isolation by a single aspiration procedure, hematopoietic and mesenchymal stem/progenitor cells may be co-transplanted, and improve the regeneration of vascular dependent tissues such as bone, muscle, adipose, dermal, nerve grafts, and may have implications in the regeneration of internal organs.

## Materials and Methods

### Isolation of hematopoietic and mesenchymal stem/progenitor cells from the same human bone marrow sample

**Figure 1:**
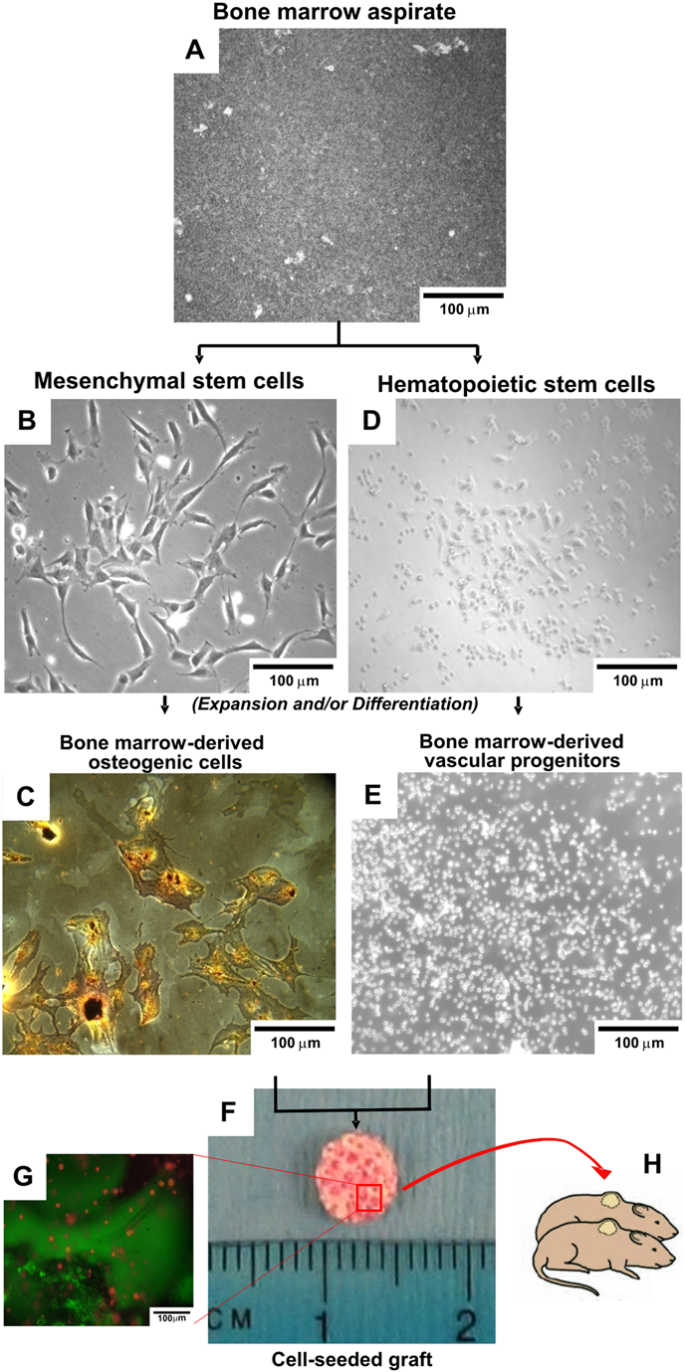
Isolation of hematopoietic stem/progenitor cells and mesenchymal stem/progenitor cells from a single bone marrow aspiration. A. Human bone marrow is aspirated from the iliac crest of donor patients. B. Mesenchymal stem/progenitor cells (MSC) isolated from human bone marrow attach to tissue culture plates and assume typical spindle, fibroblast-like shape. C. Von Kossa stained MSC-derived osteoblasts in osteogenic differentiation medium. Black stained mineralized nodules are observed as well as pericellular staining throughout the plate. D. Hematopoietic stem/progenitor cells (HSCs) are isolated from the same human bone marrow sample. E. HSCs are expanded in suspension culture, smaller than MSCs, and non-adherent, in addition to maintaining spherical shape. F. MSCs are seeded on the surfaces of the micropores of the 3D cylindrical calcium phosphate (CP) scaffold. Culture expanded HSCs with or without VEGF are then seeded in Matrigel and infused into the micropores of the 3D CP scaffolds to complete implant fabrication (controls included Matrigel with no HSCs, or with VEGF alone). G. Carboxyfluoroscein diacetate (CFDA) labeled MSC and HSCs labeled with red CM-DiI are visualized in the micropores of the 3D graft. Green MSC are on the surface of the micropores of the CP scaffold, whereas red HSCs are suspended in Matrigel that is infused into MSC-occupied pore surface. H. Scaffolds are implanted subcutaneously in the dorsum of immunocompromized mice.

Bone marrow cells were isolated from whole marrow aspirates of the iliac crest of each of three healthy male donors (AllCells, Berkeley, CA) and plated as in **Fig. 1A**. Human mesenchymal stem cells (MSC) were isolated per our previous methods using RosetteSep mesenchymal enrichment cocktail (StemCell Technologies, Vancouver, Canada) [33] (**Fig. 1B**) as mononucleated and adherent cells. In a separate experiment, MSCs were found not to express CD34 (data not shown). MSC were culture-expanded in Dulbecco's Modified Eagle's Medium (DMEM-c; Sigma, St. Louis, MO) supplemented with 10% fetal bovine serum (FBS; Atlanta Biologicals, Norcross, GA), and 1% antibiotic and antimycotic (10,000 U/mL penicillin (base), 10,000 µg/mL streptomycin (base), 25 µg/mL amphotericin B) (Atlanta Biologicals) at 37°C, 95% humidity, and 5% CO_2_ [34], [35].

A subset of the whole marrow was used to isolate CD34^+^ cells using EasySep magnetic nanoparticles (StemCell Technologies, Vancouver, Canada). The whole marrow was mixed with the CD34^+^ selection cocktail and magnetic nanoparticles per manufactureŕs protocol. Additional experiments also utilized commercially available human bone marrow derived CD34^+^ cells for verification (AllCells, Berkeley, CA). The isolated CD34^+^ cells (**Fig. 1D**) were removed from solution using a magnet (StemCell Technologies, Vancouver, Canada) and culture-expanded in IMDM (Iscovés Modified Dulbeccós Medium), supplemented with 20% FBS, 100 ng/mL SCF (stem cell factor), 100 ng/mL Flt-3 ligand, 20 ng/mL IL-3 (interleukin-3), and 20 ng/mL IL-6 in a non-tissue culture treated dish. Note that fibroblast-like MSCs (**Fig. 1B**) exhibit drastically different morphology from HSCs (**Fig. 1D**) that are rounded and smaller, when both stem/progenitor cell types were cultured *in vitro*. Isolated CD34^+^ cells were culture expanded (**Fig. 1E**) and a subset of these cells were differentiated into endothelial-like cells, with details described below.

### Osteogenic differentiation of mesenchymal stem cell-seeded bone grafts

Culture expanded bone marrow-derived MSCs were detached from culture plates using trypsin-EDTA and formed a 5×10^6^ cells/mL suspension. Ethylene oxide gas sterilized 3D-calcium phosphate (CP) scaffolds (BD Biosciences, Bedford, MA) were pre-wetted in DMEM with 10% FBS, and submerged into MSC suspension in polypropylene round bottom test tubes with snap-caps. Mineralized CP scaffolds were non-compressible sponges with hydration capacity of 30 µL, 60±10 µm porosity, and 200–400 µm pore size. The rationale for selecting CP scaffolds is primarily due to its widespread use in bone regeneration. Tubes were snap-sealed and vacuum was applied using a 20 cc syringe and incubated at 37°C for 3 hrs. MSC-seeded 3D scaffolds were cultured overnight in expansion medium and then transferred to osteogenic culture for 21 days consisting of DMEM-c supplemented with 100 nM dexamethasone, 0.05 mM ascorbic acid-2-phosphate and 10 mM β-glycerophosphate, per our previous work [34]–[36], [37]. Human MSCs cultured under osteogenic condition underwent osteogenic differentiation and mineral deposition **Fig. 1C**.

### Co-transplantation of HSCs and MSCs

Culture expanded CD34^+^ cells were suspended in Matrigel (BD Biosciences) at a density of 1×10^6^ cells/mL. Microporous CP scaffolds with MSCs seeded on pore surfaces were dried with sterile gauze, immediately submerged in the CD34^+^ cell suspended gel and subjected to mild vacuum to induce infusion of cell/gel suspension into the pores (200–400 µm) of the CP scaffold (5×3 mm^3^; dia.×height) (**Fig. 1F,1G**). Scaffolds were maintained in DMEM-c overnight prior to implantation. A total of four groups were created: 1) MSC transplantation alone, 2) VEGF-adsorbed MSC transplantation, 3) co-transplantation of MSCs and CD34^+^ cells, and 4) VEGF-adsorbed co-transplantation of MSCs and CD34^+^ cells. Microporous CP scaffolds with cytokine-free and cell-free Matrigel served as controls.

### Ectopic implantation of tissue grafts in vivo

All tissue grafts, including controls, were implanted following local IACUC approval. Nude mice were weighed and anesthetized with 3% isoflurane inhalation in an induction chamber, with anesthesia maintained with a nose cone (isoflurane 1–3%). A 2 cm-long linear incision was made along the midsagittal line of the dorsum. Tissue grafts were implanted in the subcutaneous pocket superior to dorsal muscles (**Fig. 1H**). All grafts were harvested after 4 wks by removing the fibrous capsule, and cut into two halves. One half was lysed in 1× triton-X solution, crushed, sonicated on ice for 20 s, and stored at −20°C until further analysis for ELISA, etc. The second half was fixed in 10% formalin and either embedded in GMA or paraffin for histological and immunohistochemical analyses as described below.

### Histology, immunohistochemistry, histomorphometry, and biochemical analyses

Specimens were demineralized in equal volumes of 20% sodium citrate and 50% formic acid, subsequently embedded in paraffin, sectioned in the transverse plane at 5 µm thickness and stained with hematoxylin and eosin (H&E) or Massońs Trichrome stain [35], [39], [41]. Undemineralized specimens were embedded in GMA, sectioned at 20 µm and stained with H&E [42]. Sequential sections were immunostained for human osteocalcin (Cambridge, MA) and human nuclei (Millipore, Billerica, MA) for visualizing the extent of osteogenesis and the contribution of transplanted cell to neovascularization, respectively. Computerized histomorphometric analysis was performed to quantify blood vessel number and blood vessel diameter using grid analysis [43]. All biochemical assays were evaluated using thawed, lysed samples. DNA content was determined using fluorescent DNA quantification kit (BioRad Labs, Hercules, CA) and expressed as ng DNA per mL of sample. Osteocalcin was detected using a human osteocalcin ELISA kit (R&D Systems, Minneapolis, MN) and von Willebrand factor (vWF) using human vWF-specific ELISA (Helena Laboratories, Beaumont, TX).

### Endothelial differentiation of hematopoietic stem/progenitor cells in vitro

Culture expanded HSCs were induced to differentiate into endothelial-like cells by plating onto fibronectin coated plates with endothelial differentiation medium containing IMDM supplemented with 10% FBS, 1% Antibiotics/Antimycotics, 10 ng/mL VEGF (vascular endothelial growth factor), 1 ng/mL bFGF (basic fibroblast growth factor), and 2 ng/mL IGF-1 (insulin-like growth factor-1).

#### Statistical analysis

ANOVA and post-hoc Bonfferroni tests were used to compare all quantitative data between the control group and each experimental group at an α level of 0.05.

## Results

### In vivo vascularization of tissue grafts generated from CD34^+^ hematopoietic stem/progenitor cells and CD34^−^ mesenchymal stem/progenitor cells

**Figure 2:**
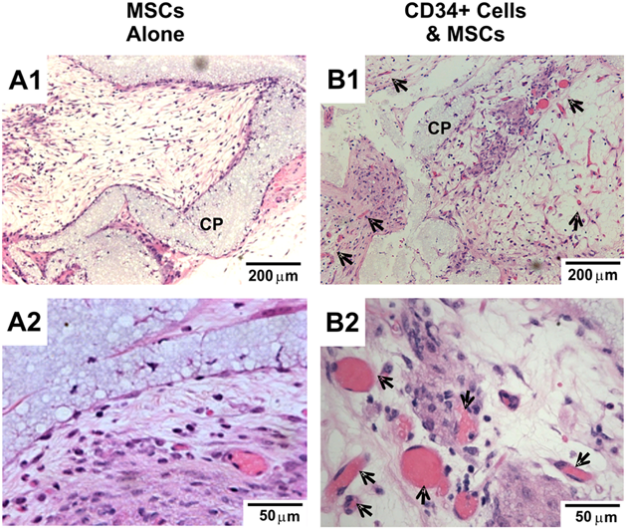
Vascularization of *in vivo* implanted tissue grafts. H&E staining. A. Microporous calcium phosphate (CP) scaffolds seeded with mesenchymal stem/progenitor cells (MSCs) alone showed minimal vascularization of the micropores of the implant. B. Co-transplantation of hematopoietic stem/progenitor cells (HSCs) and MSCs resulted in substantial numbers of blood vessels (black arrows) in the micropores of the CP scaffolds.

Microporous, 3D calcium phosphate scaffolds co-transplanted with HSCs and mesenchymal stem/progenitor cells implanted subcutaneously in the dorsum of athymic nude mice were evaluated for both angiogenesis and osteogenesis. Four weeks after *in vivo* implantation, bone grafts with co-transplanted HSCs and MSCs demonstrated notably visible vascular ingrowth into the micropores of CP scaffolds (black arrows in **Fig. 2B1, B2**). In comparison, only limited vascular ingrowth was observed in MSC transplantation alone (**Fig. 2A1, A2**). Neovascularization was apparently anastomosed with host vasculature, given the presence of red blood cells within vessel walls formed by endothelial cells.

### Endothelial differentiation of hematopoietic stem/progenitor cells

**Figure 3:**
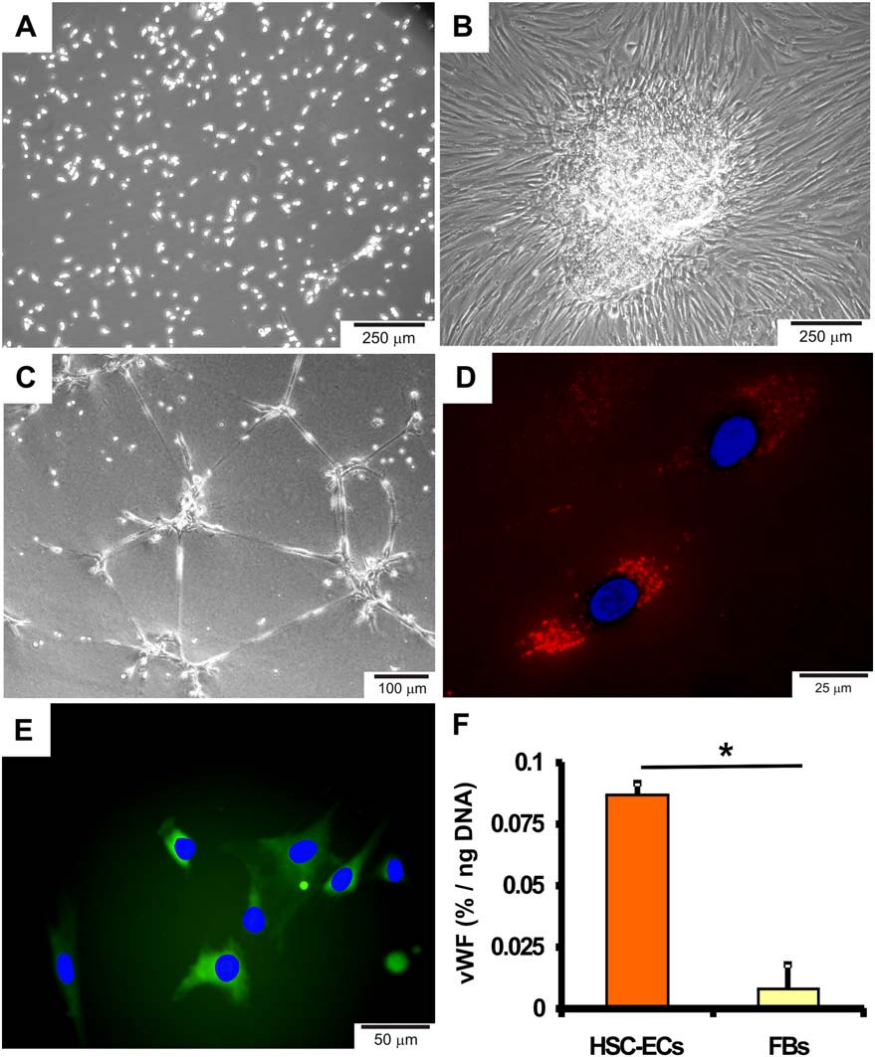
Endothelial differentiation of HSCs *in vitro*. A. Hematopoietic stem/progenitor cells (HSCs) propagated in suspension culture, assuming spherical shape. B. HSCs form endothelial-like colonies in fibronectin-coated plates. C. Formation of tubular intercellular structures in 3D Matrigel culture. Uptake of acetylated-LDLs (red) (blue: DAPI) (D) and vWF (von Willebrand Factor) immunofluorescent stain (green) (E). F. Quantification of vWF measured by ELISA showing substantial expression of vWF in HSC-derived endothelial-like cells, in comparison with dermal fibroblasts as controls.

We further examined whether CD34+ cells isolated in the present study can be differentiated into endothelial progenitor cells or endothelial cells by VEGF, a growth factor with potency on endothelial differentiation [15]. We first isolated CD34^+^ cells from donated human bone marrow samples by positive selection (**Fig. 3A**). When grown in suspension culture, CD34^+^ cells remained spherical and non-attached (**Fig. 3A**). Upon seeding on fibronectin-coated plates in endothelial differentiation medium including VEGF, CD34^+^ cells attached to the plate and formed colonies (**Fig. 3B**). When seeded on 3D Matrigel and exposed to endothelial differentiation medium, CD34^+^ cells formed interconnecting tubular networks that are reminiscent of early angiogenesis and characteristic of endothelial progenitor cells (**Fig. 3C**). Endothelially differentiated CD34^+^ cells on fibronectin-coated plates showed positive immunofluorescent staining for acetylated LDLs (**Fig. 3D**) and von Willebrand factor (vWF) (**Fig. 3E**), both typical for endothelial progenitor cells. Quantitatively, endothelially differentiated CD34^+^ cells (HSC-ECs) expressed substantial vWF normalized to DNA content, in comparison to dermal fibroblasts that were used as controls (**Fig. 3F**).

### Enhanced angiogenesis of VEGF-stimulated, co-transplanted HSC and MSC co-seeded grafts in vivo

**Figure 4:**
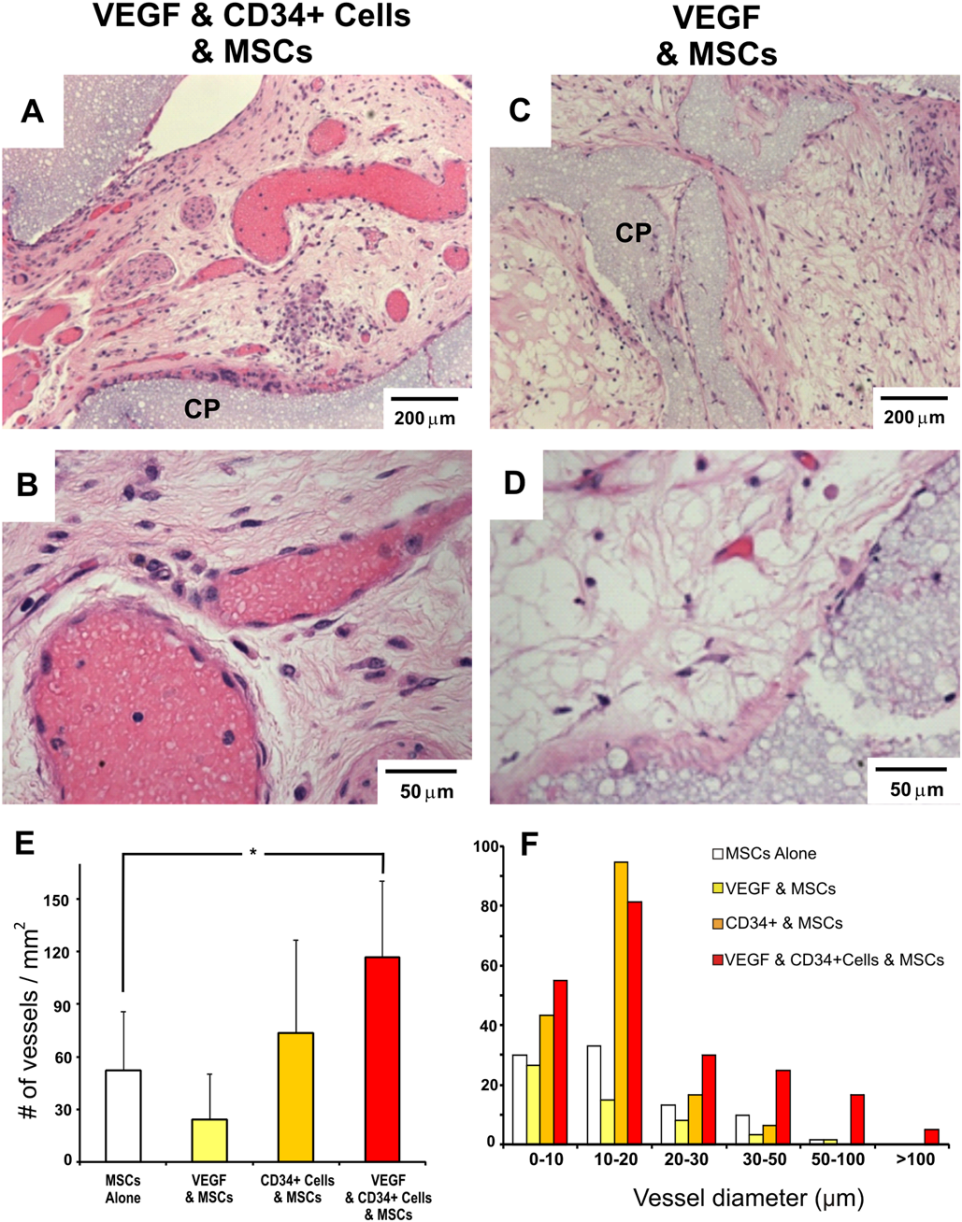
VEGF enhances neovascularization of HSC and MSC co-transplanted scaffolds. A. VEGF delivered with transplanted HSCs promoted increased neovascularization of large diameter, indicative of increasingly mature blood vessels. Red blood cells are observed in neovasculature indicating anastomosis to host and functionality (A–D:H&E stain). B. Higher magnification of A. C. VEGF delivered MSC transplantation group (no transplanted HSCs) fails to show substantial neovascularization as demonstrated by few sparse and small blood vessels. D. High magnification of C. CP: calcium phosphate. E. Quantification of the average number of vessels showing significantly increased vasculature in VEGF-stimulated HSC and MSC co-transplantation group (n = 5, p<0.05). F. Histogram presentation of the average vessel diameters show that VEGF delivery resulted in larger, and likely more mature, blood vessels towards the right tail of the histogram.

Four weeks after *in vivo* implantation, bone grafts with VEGF-stimulated, co-transplanted HSCs and MSCs showed substantial vascular ingrowth into the micropores of CP scaffolds (**Fig. 4A, 4B**). In comparison with VEGF-delivered MSC transplantation alone (**Fig. 4C, 4D**) or co-transplanted HSCs and MSCs but without VEGF delivery (**Fig. 2B1, 2B2**), VEGF-stimulated HSC and MSC co-transplantation yielded not only more, but also larger blood vessels that were populated by red blood cells and lined by endothelial cells (**Fig. 4A, 4B**). This is especially true when contrasted with VEGF-MSC sample in **Fig. 4C, 4D**, consistent with previous findings that VEGF delivery alone fails to elaborate mature blood vessels [44], [45]. Quantitatively, the average number of blood vessels markedly increased for VEGF-stimulated co-transplantation of HSCs and MSCs, in comparison with MSC transplantation alone, VEGF delivery alone and co-transplantation of HSCs and MSCs but without VEGF delivery (**Fig. 4E**). VEGF delivery with HSC and MSC co-transplantation yielded an ∼240% increase in vessel number over MSC transplantation group (p<0.05) (**Fig. 4E**). VEGF delivery along with co-transplantation of HSCs and MSCs also yielded large blood vessels, in comparison with MSC transplantation alone, VEGF delivery alone or co-transplantation of HSCs and MSCs but without VEGF (**Fig. 4F**).

### Contribution of transplanted human HSCs and MSCs to endothelium

**Figure 5:**
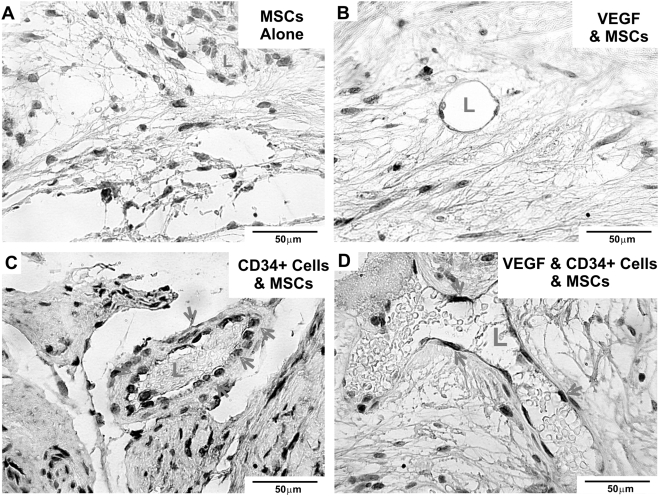
Transplanted human HSCs and MSCs engraft *in vivo* and into vascular endothelium of host vasculature. Immunostaining (brown) of human specific nuclei of tissue grafts by MSC transplantation alone (without HSCs) (A), MSC transplantation with exogenous VEGF (B), co-transplantation of MSCs and HSCs without VEGF delivery (C), or co-transplantation of MSCs and HSCs with VEGF delivery (D). Red arrows point to human nuclei that engraft to forming blood vessel wall surrounding functional lumen (L) filled with red blood cells.

Transplanted HSCs and MSCs were engrafted into the endothelium of host-derived blood vessels. Upon immunohistochemical visualization of human-specific nuclear antibody, transplanted human HSCs and MSCs were found broadly in tissue grafts and in some cases, formed vascular endothelium with or without VEGF delivery (red arrows in **Fig. 5C,5D**). Engraftment of transplanted human cells and co-localized host (mouse) cells participated in the formation of endothelium (**Fig. 5C,5D**). Red blood cells populated blood vessel lumen (L in **Fig. 5C,5D**) that was lined by human-host (mouse) derived endothelium, suggesting that blood vessels in tissue grafts anastomosed with host vasculature. In contrast, blood vessels formed by MSC transplantation alone with or without VEGF, but in the absence of transplanted HSCs, had broad engraftment of human cells, but rarely within the endothelium (**Fig. 5A, 5B**).

### Ectopic mineralization in vascularized grafts generated from CD34^+^ and mesenchymal stem/progenitor cells

**Figure 6:**
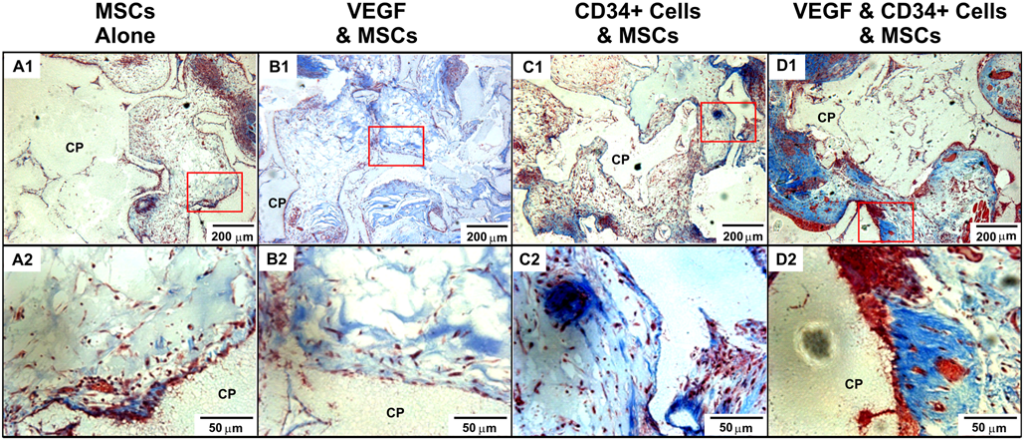
Collagen apposition in tissue grafts *in vivo*. A–D. Masson's trichrome staining (blue) shows increased pre-mineralizing collagen deposition and osteoid formation in co-transplantation of MSCs and HSCs without VEGF (C1) and VEGF-delivered MSC and HSC co-transplantation sample (D1), in contrast to MSC transplantation alone (A1) and VEGF-delivered MSC transplantation sample (B1). A2–D-2. Magnification of red boxes in A1–D1, respectively.

**Figure 7:**
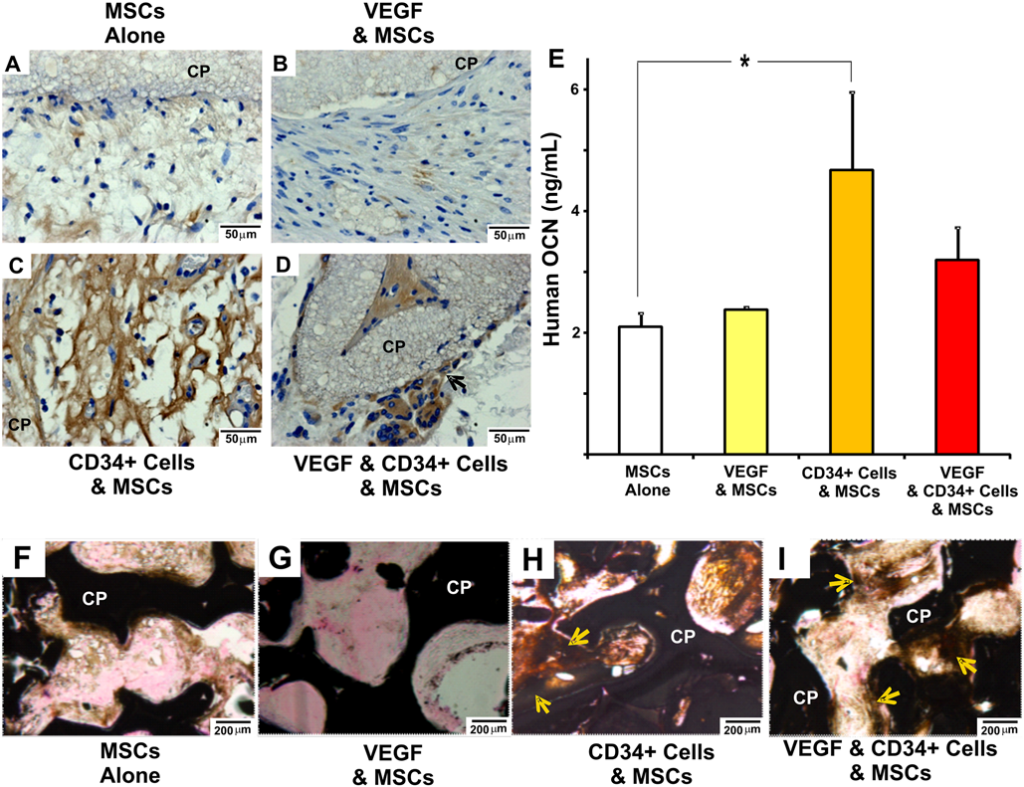
Expression of human osteocalcin and mineral deposition in tissue grafts *in vivo*. Human osteocalcin immunostaining displays increased levels of expression in MSC and HSC co-transplantation sample (C) and VEGF-delivered MSC and HSC co-transplantation sample (D), in contrast with MSC transplantation alone sample (A) and VEGF-delivered MSC transplantation sample (B). Osteoblast-like cells are observed lining the calcium phosphate scaffold (CP) (black arrow in D). E. Quantified human osteocalcin content confirms significantly increased human osteocalcin expression of the co-transplantation group of MSCs and HSCs (n = 5, p<0.05). Interestingly, VEGF delivery decreased human osteocalcin expression, despite the higher level of vascularization (see detailed discussion in text). F–I. Undecalcified H&E stained sections of in vivo implanted scaffolds show increased dark mineralized tissue throughout the co-transplanted MSC and HSCs cell-seeded scaffold (yellow arrows in H) and VEGF-delivered, co-transplanted MSC and HSC-seeded scaffold (yellow arrows in I) groups, in contrast to MSC transplantation alone (F) and VEGF-only (G).

Ectopic mineral apposition was observed in the micropores of the CP grafts. Massońs Trichrome staining showed areas of collagen fiber accumulation within the micropores of the CP scaffold (**Fig. 6**). While MSC transplantation with or without VEGF delivery resulted in mild areas of Masson's trichrome staining (**Fig. 6A1, A2 and Fig. 6B1, B2**), moderate collagen apposition was found in association with co-transplanted HSCs and MSCs but without VEGF (**Fig. 6C1, C2**). Importantly, substantial Masson's trichrome staining was present along the interface of tissue formation and microporous CP scaffolds (**Fig. 6D1**). High magnification of VEGF-delivered, HSC and MSC co-transplantation sample showed robust collagen deposition and areas of apparent bone trabecula-like structures (**Fig. 6D2**). Immunohistochemical analysis showed the presence of human-specific osteocalcin in the micropores of CP scaffolds implanted subcutaneously in SCID mice (**Fig. 7A–E**), indicating that transplanted human cells synthesized osteocalcin. Isolated areas of human-specific osteocalcin staining were found in MSC transplantation with or without VEGF delivery (**Fig. 7A,B**). In contrast, representative samples of co-transplantation of MSCs and HSCs with or without VEGF showed substantial areas of human-specific osteocalcin staining (**Fig. 7C,D**). Osteoblast-like cells are observed on the surface of calcium phosphate scaffolds (CP) (black arrow in **Fig. 7D**). Quantitatively, the expression of human-specific osteocalcin was significantly more robust upon co-transplantation of MSCs and HSCs, interestingly, without VEGF delivery, than MSC transplantation alone, presenting an ∼220% increase (p<0.05) (**Fig. 7E**). Mineral apposition is verified on undecalcified sections (yellow arrows in **Fig. 7F–I**). Isolated brown areas at the interface of newly formed tissue and CP scaffold were found in MSC transplantation with or without VEGF delivery (**Fig. 7F,G**). In contrast, extensive brown areas were present in the co-transplantation sample seeded with HSCs and MSCs (yellow arrows in **Fig. 7H**). Similarly, the representative sample of co-transplantation of HSCs and MSCs with VEGF delivery also showed extensive areas of mineral apposition (yellow arrows in **Fig. 7I**).

## Discussion

The present results constitute an original discovery that tissue vascularization and regeneration is enhanced by co-transplantation of mesenchymal stem/progenitor cells and hematopoietic stem/progenitor cells. Given that HSCs and MSCs can be readily isolated from a single bone marrow aspiration procedure [46]–[48], the significance of our data is to promote a translational approach to combine the delivery of HSCs and MSCs towards generating vascularized tissues. Given that suboptimal angiogenesis is the common roadblock in tissue regeneration, the present co-transplantation of HSCs and MSCs offers an alternative to other angiogenic approaches that have been previously investigated, such as delivery of growth factors or the fabrication of blood vessel analogs [3]–[6]. MSCs and HSCs have rarely been delivered in combination for the healing of defects or the treatment of diseases, partially due to separate communities in which HSCs and MSCs are studied. If the present approach is extendable to the promotion of vascularization of other tissues such as adipose, cardiac, muscular, nerve and dermal grafts, then a single bone marrow aspirate may provide threshold numbers of expandable multi/pluri-potent stem/progenitor cells including MSCs and HSCs for vascularized tissue regeneration [37], [38], [40], [49]–[52]. An additional advantage of combined delivery of HSCs and MSCs appears to be the disadvantages in association with the cost of multiple angiogenic growth factors, transplantation of endothelial cells that are difficult to isolate from patients and difficult to expand in vitro, and challenges associated with microsurgery for connecting bioengineered blood vessels.

The clear advantage of increased vascular number and vascular diameter by co-transplantation of MSCs and HSCs, over MSC transplantation alone, appears to indicate several putative pathways via which HSCs may participate in synergistic actions with MSCs. First, transplanted HSCs in the present work differentiate into endothelial cells *in vitro*, and engraft into host-derived vasculature *in vivo*. These findings suggest that HSCs not only possess the critical signaling potential during tissue repair, but also may directly participate as repair cells. HSCs may differentiate into various hematopoietic elements, and anastomose with host-derived vasculature. The incorporation of transplanted human cells in vascular endothelium suggests that vascular signaling by transplanted human cells promotes anastomosis with host vasculature. Additional studies can be designed to separately tag HSCs and MSCs, so to appreciate the relative contribution of both cell types to the neovasculature. Second, MSCs and HSCs may act as each other's supportive cells, and reciprocally promote tissue-forming and vascular support functions. This speculation is clearly beyond the scope of the present study, but warrants separate studies. Third, we are somewhat surprised that MSC transplantation alone in the present work yields somewhat disappointing regeneration as well poor angiogenesis. This may be attributed to a modest number of MSCs and partial differentiation of MSCs into osteogenic cells. Also, our data do not rule out a possibility that MSCs have differentiated into endothelial-like cells in vivo in the present model, as shown before [53]–[55]. Human nuclei staining does demonstrate engraftment of the transplanted human cells in regenerating tissue in the pores of CP scaffold, but few transplanted MSCs are found in the vascular wall unless HSCs are co-transplanted. Follow up studies are warranted to determine to what extent MSCs differentiate into endothelial cells *in vivo*.

Vascular endothelial growth factor (VEGF) is used in the present work along with co-transplantation of HSCs and MSCs, and further enhances vascular number and vascular diameter. In the present complex system, VEGF's actions are likely multi-dimensional. VEGF clearly promotes the differentiation of HSCs towards endothelial progenitor cells or endothelial cells in vitro as shown in the present work, but may have also concomitantly signaled and recruited host-derived vascular network in vivo [56]. VEGF and other angiopoietins mobilize and activate hematopoietic cells, and may provide obligatory signaling for the differentiation and stabilization of endothelial cells [57], [58]. Differentiated endothelial cells express abundant VEGF receptors, but secrete little VEGF [59], whereas VEGF stimulates autocrine pathways of HSCs and promotes cell survival [60]. Thus, the present approach to co-transplant HSCs and MSCs may maximize the potential of transplanted multi-lineage cells, and allow them to be activated by local cues of the injured tissue. The present VEGF delivery via diffusion from a scaffold gel will lead to its rapid release, consistent with previous work advocating that VEGF should be delivered early in regeneration [7]. During native angiogenesis, VEGF expression peaks early, followed by other angiopoietins such as PDGF (platelet derived growth factor) [7]. Given that HSCs are progenitors of platelets, we speculate that the transplanted CD34^+^ cells may directly differentiate into or mediate the differentiation of platelets which are important for vessel wall maturation and the recruitment of mural cells [61].

An interesting observation of our data is that VEGF delivery is accompanied by a decline of human osteocalcin content. This is likely attributed to several factors. A fraction of CD34^+^ cells have been shown to express osteocalcin and to engraft in healing fractures [62]–[64]. Also, a percentage of osteocalcin and alkaline phosphatase expressing cells from peripheral blood with osteogenic potential are CD34 positive [65]. In the present work, CD34^+^ cells may have participated in mineral apposition and the expression of human osteocalcin. Conversely, delivery of VEGF in the present work may have promoted endothelial differentiation of the transplanted CD34^+^ cells; accordingly, fewer CD34^+^ cells engage in osteocalcin synthesis. Additional studies are designed to explore the effects of VEGF dosing on HSCs and MSCs. Together, the present co-transplantation of hematopoietic and mesenchymal stem/progenitor cells yields vascularized tissue regeneration. Vessel number and diameter that are elaborated by HSCs and MSCs are found to further improve upon VEGF delivery. These original findings are perhaps reminiscent of the native development process as well characterized in bone development. Prior to the arrival of primary ossification center artery, diaphyseal bone fails to develop. Although the oxygenation and diffusion properties of the engineered bone are not outcome measures of the present study, we suspect that the present approach may have induced angiogenesis and vasculogenesis both from outside in (host-derived) and inside out (human cell driven). Given the approximate 100–200 µm limitation for vascular supply in native tissue, it would be of interest to determine whether oxygenation and diffusion properties of engineered bone, such as the presently derived, differ from native tissue. The present approach likely filters out some end lineage cells from bone marrow aspiration and provide an initial selection of HSC and MSC populations. Therefore, this approach differs from whole marrow transplantation with or without further processing. Taken together, synergistic actions of hematopoietic and mesenchymal stem/progenitor cells may provide an alternative approach for the regeneration of vascular tissues such as bone, adipose, cardiac, muscle and dermal grafts.
